# Rice-Infecting *Pseudomonas* Genomes Are Highly Accessorized and Harbor Multiple Putative Virulence Mechanisms to Cause Sheath Brown Rot

**DOI:** 10.1371/journal.pone.0139256

**Published:** 2015-09-30

**Authors:** Ian Lorenzo Quibod, Genelou Grande, Eula Gems Oreiro, Frances Nikki Borja, Gerbert Sylvestre Dossa, Ramil Mauleon, Casiana Vera Cruz, Ricardo Oliva

**Affiliations:** 1 Plant Breeding, Genetics, and Biotechnology Division, International Rice Research Institute, Los Baños, Philippines; 2 T.T. Chang- Genetic Resources Center, International Rice Research Institute, Los Baños, Philippines; 3 Department of Phytomedicine, Leibniz Universität Hannover, Hannover, Germany; Virginia Tech, UNITED STATES

## Abstract

Sheath rot complex and seed discoloration in rice involve a number of pathogenic bacteria that cannot be associated with distinctive symptoms. These pathogens can easily travel on asymptomatic seeds and therefore represent a threat to rice cropping systems. Among the rice-infecting *Pseudomonas*, *P*. *fuscovaginae* has been associated with sheath brown rot disease in several rice growing areas around the world. The appearance of a similar *Pseudomonas* population, which here we named *P*. *fuscovaginae*-like, represents a perfect opportunity to understand common genomic features that can explain the infection mechanism in rice. We showed that the novel population is indeed closely related to *P*. *fuscovaginae*. A comparative genomics approach on eight rice-infecting *Pseudomonas* revealed heterogeneous genomes and a high number of strain-specific genes. The genomes of *P*. *fuscovaginae*-like harbor four secretion systems (Type I, II, III, and VI) and other important pathogenicity machinery that could probably facilitate rice colonization. We identified 123 core secreted proteins, most of which have strong signatures of positive selection suggesting functional adaptation. Transcript accumulation of putative pathogenicity-related genes during rice colonization revealed a concerted virulence mechanism. The study suggests that rice-infecting *Pseudomonas* causing sheath brown rot are intrinsically diverse and maintain a variable set of metabolic capabilities as a potential strategy to occupy a range of environments.

## Introduction

The increasing global trade activities are the main cause of movement of plant pathogens that continue to threaten modern agriculture. In such scenario, pathogen populations tend to diversify and increase their evolutionary potential as they encounter more favorable conditions and recombination opportunities [1]. This is particularly true for many plant pathogens that colonize the rice (*Oryza sativa* L.) seed, which include members of the genus *Pseudomonas*. One of the most common rice-infecting pathogens is *Pseudomonas fuscovaginae* (*Pfv*), a seed-borne and seed-transmitted Gram-negative bacterium that causes sheath brown rot and grain discoloration. Several reports have demonstrated epiphytic and endophytic colonization of *Pfv* in symptomless rice seeds [2–4], suggesting that gene flow is still effective and more research is needed to understand pathogen diversity and distribution. This pathogen was first reported in Hokkaido, Japan in 1976 [2, 5] and soon appeared in many tropical and subtropical rice-growing regions of the world i.e. Latin America [6, 7], Sub-Saharan Africa [8–10], Southeast Asia [11–13], and Australia [14].

Under favorable low temperature conditions, *Pfv* colonizes the rice sheath, producing brown to reddish brown necrotic lesions. If conditions persist, the lesions can progress toward the panicle, causing seed discoloration and grain sterility [6, 7]. The intrinsic capability of *Pfv* in colonizing multiple plant tissues as well as its ability to survive as an epiphyte on the seed surface [3] or endophytically in roots, stems, and leaves [4] probably require quite a versatile metabolism. In addition, *Pfv* has a broad host range among wild and cultivated grasses [3, 15, 16], which also reveals a diverse panel of pathogenicity factors that is worth exploring.

From a taxonomical perspective, the genus *Pseudomonas* comprises at least two main lineages, the *P*. *aeruginosa* lineage and the *P*. *fluorescens* lineage. Phylogenetic analysis of 16S rRNA, *gyrB*, *rpoB* and *rpoD* sequences clearly associates *Pfv* with the *P*. *fluorescens* subgroup members [17]. This group appears to be highly diverse, harboring unusual levels of intra-species heterogeneity. Silby and co-workers [18] compared three *P*. *fluorescens* genomes (SBW25, Pf0-1, and Pf-5) and found that only 61% of genes were shared among them. Subsequent studies also found high numbers of unique genes when comparative analysis was performed among different genomes of *P*. *fluorescens* [19–21].

Apart from *Pfv*, other rice-infecting *Pseudomonas* pathogens have been isolated from plant tissues showing sheath brown rot symptoms. In the Philippines, disease surveys on tropical rice ecologies have isolated fluorescent *Pseudomonas* colonies similar to *Pfv* [12]. In 1998, two of these colonies, named IRRI 6609 and IRRI 7007, were isolated from rice sheaths collected in Davao and Palawan regions in the month of December when temperatures are usually less than 20°C. Also, the strain S-E1 was isolated in an agronomy trial in Siniloan, Luzon Island during a low temperature spell [12, 22]. Serological, biochemical, and genetic analyses of IRRI 6609, IRRI 7007, and S-E1 together with other *Pseudomonas* groups revealed that all three strains were closely related to *Pfv* but were part of a distinct population [12, 22]. Until the taxonomic status of these populations is clarified, we will designate these populations as *P*. *fuscovaginae*-like (*Pfv*-like).

It is well documented that plant pathogenic bacteria evolved a plethora of mechanisms to modulate host environment in order to facilitate colonization [23–25]. Several secretion systems are used to deliver molecules that interact with apoplastic or cytoplasmic plant components [26]. While many Gram-negative bacteria inject type III (T3) effector proteins into host cells [25, 27], other key virulence factors are also widely used such as cell wall-degrading enzymes (CWDEs), phytotoxins, extracellular polysaccharides, and phytohormones, among others [24]. However, only few studies have really addressed the presence of pathogenicity factors in rice-infecting *Pseudomonas*. For instance, the two N-acyl homoserine lactone (AHL) quorum sensing systems (PfsI/R and PfvI/R) of *Pfv* have been reported by Mattiuzzo *et al*. [28] to be involved in virulence. The presence of syringotoxin in *Pfv* extracts [29, 30] was highlighted as a virulence factor when Batoko and co-workers [31] showed that it may affect plant membrane integrity by inhibiting a membrane-associated H^+^-ATPase *in vitro*. Recently, a *Pfv* mutant screening on *Chenopodium quinoa* and rice identified additional virulence factors involved in adhesion, phytotoxins, and secretion [32]. At the moment, it is not clear whether other factors may be relevant during the *Pseudomonas*–rice interaction.

The fact that other *Pseudomonas* populations are able to infect rice opens the door for comparative analysis to identify a common set of virulence factors and depict the evolutionary context of this group. To better explore *Pfv*-like genomic composition, structure, and diversity, we obtained shotgun sequences of the two strains from the Philippines (IRRI 6009 and IRRI 7007) and compared their genome assemblies with draft genomes of several *Pfv* and *Pfv*-like strains that were recently released [33–35]. We found that *Pfv*-like strains are closely related to *Pfv*. Although sequenced strains represent only a fraction of the overall diversity, we showed that *Pfv* as well as *Pfv*-like populations are not genetically homogeneous, having acquired high levels of diversification. We redefined the understanding of the *Pfv* pan-genome and identified a set of common virulence factors that may be important to successfully colonize rice sheath. Interestingly, *Pfv* and *Pfv*-like have plastic genomes with a high proportion of strain-specific genes and unique metabolic capabilities. We also defined the core secretome for *Pfv* and *Pfv*-like, which showed strong signatures of positive selection that matched with both pathogen lifestyles.

## Materials and Methods

### Pathogenicity test

The pathogenicity of *Pfv*-like strains IRRI 6609, IRRI 7007, and S-E1 were performed on the rice cultivars Azucena and Moroberekan using the toothpick method at maximum tillering stage (40–45 days after transplanting). Bacterial cultures were grown for 24 h at 28–30°C on King’s medium B (KB) and suspended in sterile demineralized water [22]. The middle portion of the sheath was pricked with the toothpick dipped in the bacterial suspension with 10^8^ cfu/ml. Plants inoculated with sterile demineralized water served as negative control. The inoculated plants were incubated in a growth chamber at 23°C/18°C day/night temperature with 90% relative humidity and photoperiod 12h/12h (light/dark). Sheath discoloration was observed at 14 days post inoculation (dpi) and, to further confirm the disease, the inoculated plants were kept until maturity stage for observations of grain discoloration.

### *Pseudomonas* genomes and whole-genome alignments

We used different sources to obtain a representative sample of *Pfv*-like genomes. The genome sequence of the strain S-E1 was downloaded from GenBank [35]. *Pfv*-like cultures of IRRI 6609 and IRRI 7007 were grown overnight at 28°C. Isolation of genomic DNA was done using Easy-DNA kit (Invitrogen, USA) following the manufacturer’s protocol. Genome sequencing was contracted as service to BGI-Shenzhen (Shenzhen, China), producing 90-bp paired-end reads using Illumina GAIIx technology. Filtered paired-end reads were *de novo* assembled into contigs and scaffolds using CLC Genomics Workbench 6.5 (CLC bio, Denmark). Alternative assemblies did not result in better outputs so we followed CLC. Gene calling and annotation were performed using JGI/IMG-ER 4 [36]. The genome sequence of the five *Pfv* strains UPB0736 (Madagascar), CB98818 (China), ICMP 5940 (Japan), and DAR 77795 and DAR 77800 (Australia) were downloaded from GenBank [33–35]. All *Pfv* (5) and *Pfv*-like (3) genes were classified according to cluster of orthologous groups (COG) terms [36]. The datasets of the strains ICMP 5940, DAR 77795, and DAR 77800 were re-annotated using RAST [37]. Predicted genes with sizes less than 50 bp were removed from the analysis. Draft genome sequences of *Pfv*-like strains IRRI 6609 and IRRI 7007 were deposited at GenBank under accession numbers JSYZ00000000 and JTBY00000000. For comparative analysis, we used a set of 79 different *Pseudomonas* genomes comprising main lineages (S1 Table). Intact prophage prediction and annotation were obtained using the PHAST server [38]. To assess if the prophage is complete or not, we calculated the number of bases, genes, and cornerstone genes and detected the presence of phage-like genes. All intact prophage should have at least a score of 90 by PHAST standards. For secondary metabolite cluster prediction, the antiSMASH 2.0 [39] website was used. In addition, selected genes were BLASTp against the *Pseudomonas* dataset with E-values less than 1e-20 and with a minimum identity of 20%. These were then visualized using CodaChrome 1.1 [40]. Whole-genome alignment was performed in two steps: first, we reordered contigs of *Pfv*-like and *Pfv* genomes against IRRI 6609 using MAUVE 2.3.1 [41] under default parameters. Locally collinear blocks and iterative alignment were arranged using MUSCLE [42]. In the second step, we produced the final alignments of the eight strains with BLASTn and visualized with BLAST Ring Image Generator 0.95 (BRIG) [43]. All alignments had a threshold E-value ≤ 1e-25.

### Average nucleotide identity (ANI) and tetranucleotide frequency correlation coefficients (TETRA) analysis

To determine the relatedness among *Pfv* and *Pfv*-like strains, average nucleotide identity (ANI) and tetranucleotide frequency correlation coefficients (TETRA) analyses were done using the JSpecies 1.2.1 [44] software under default parameters. For the ANI values, alignment calculation was done using the MUMmer algorithm [45]. The ANI and TETRA matrices were used to construct a pairwise relationship. The heatmap.2 function from the R package gplots was used to build the dendrograms and heatmaps. Furthermore, 79 *Pseudomonas* species (S1 Table) were compared for ANI using *Pfv*-like IRRI 6609 as the reference strain. The percentage value cut-off for the ANI and TETRA analyses were >95% and >99%, respectively.

### Orthologous gene identification and average amino acid identity (AAI) analysis

Orthologous gene clustering of protein and nucleotide sequences collected from the *Pfv* and *Pfv*-like genomes was performed using GET_HOMOLOGUES [46]. Pairwise alignment was done using BLASTall [47] among sequences with a minimum E-value of 1e-5. For the orthologous gene identification, OrthoMCL [48] and COGtriangles [49] algorithms were used to filter and cluster the BLAST results with sequences having a coverage of at least 50% and a minimum identity of 50%. Genes that were not positive for both algorithms were filtered out from the final list. Following this approach, we used all BLAST results to compute the average amino acid identity (AAI) for each comparison and to build the pairwise relationship as stated above.

### Secretome prediction and positive selection analysis

The secretome of *Pfv* and *Pfv*-like was predicted using 50,066 open reading frames obtained from eight genomes. The presence of secretion signal was predicted using SignalP 4.1 [50] and further filtered for transmembrane domain-containing proteins using TMHMM 2.0 [51]. To predict type III effectors, we used BLASTp against T3DB resource [52]. All the predicted secreted proteins were clustered using GET_HOMOLOGUES [46] with the same parameters mentioned above. The core genes were then extracted for exhaustive Gene Ontology (GO) term search using BLAST2GO [53] and through the Pfam 27.0 [54] and InterPro 48.0 [55] websites. Multiple alignment of the core orthologous genes were generated using TranslatorX [56] with the guidance of the MUSCLE program for the translated protein sequences. Gblocks [57] was then applied to remove spurious alignments. An estimation of the numbers of synonymous (Ks) and nonsynonymous (Ka) substitutions per site was used as parameters to assess selection at molecular level [58, 59]. The higher the ratio (ratio ω = Ka/Ks > 1), the stronger the signal of positive selection between two DNA-coding sequences. Ka/Ks ratios were calculated with the support of KaKs-Calculator 2.0 [58]. The Yn00 model [59] was used for determining the ω values.

### Phylogeny based on MLSA

The phylogenetic relationship was evaluated using concatenated nucleotide sequences of *rpoB* and *rpoD* genes (S2 Table). Multiple alignment was then performed in MEGA6 [60] using the implementation of the MUSCLE algorithm and with the help of Gblocks to secure the conserved blocks of the alignment [57]. Maximum Likelihood (ML) analysis was carried out to infer the evolutionary relationship of eight *Pfv* and *Pfv*-like strains and 26 closely related *Pseudomonas* species. The *E*. *coli* K-12 sub-strain MG1655 was used as outgroup. The ML tree was constructed using RAxML [61], with 1000 bootstrap replicates and GTRGAMMA as the substitution model. FigTree 1.4.0 (http://tree.bio.ed.ac.uk/software/figtree/) was used to visualize the tree. To strengthen the phylogenetic analysis, we used concatenated nucleotide sequences of 10 housekeeping genes: *acsA*, *aroE*, *dnaE*, *guaA*, *gyrB*, *mutL*, *ppsA*, *pyrC*, *recA*, and *rpoB* (S2 Table) on a subsample of species.

### Transcript accumulation of *Pfv*-like genes

The rice cultivar Azucena and the *Pfv*-like strain IRRI 7007 were used in a time course gene expression experiment. At maximum tillering, the leaf sheath of rice was syringe-infiltrated with *Pfv* inoculum and sampling points 0, 3, 24, 48, and 72 hours post infection (hpi) were considered. IRRI 7007 was chosen because of its high virulence. For semi-quantitative RT-PCR, the total RNA from the treated rice sheath tissues of Azucena, as well as tissues from mock samples, were obtained using the Trizol method (Invitrogen, USA). Complementary DNA (cDNA) was synthesized from the pooled RNA molecules using SuperScript III and random hexamers (Invitrogen, USA). Primers were designed to target the coding sequences of candidate pathogenicity genes (S3 Table). In addition, two core random genes were selected. RT-PCR was done with the gene-specific primers in a 20ul reaction mix and was performed in a thermal cycler machine (G-storm GS1). We used the 16S gene to normalize the *Pfv*-like gene expression assessment. This experiment was done in two independent biological replicates with three technical replicates per sample. RT-PCR products were visualized on 1.25% agarose gels.

## Result and Discussion

### *Pfv*-like populations are able to infect rice sheath and cause seed discoloration

**Fig 1:**
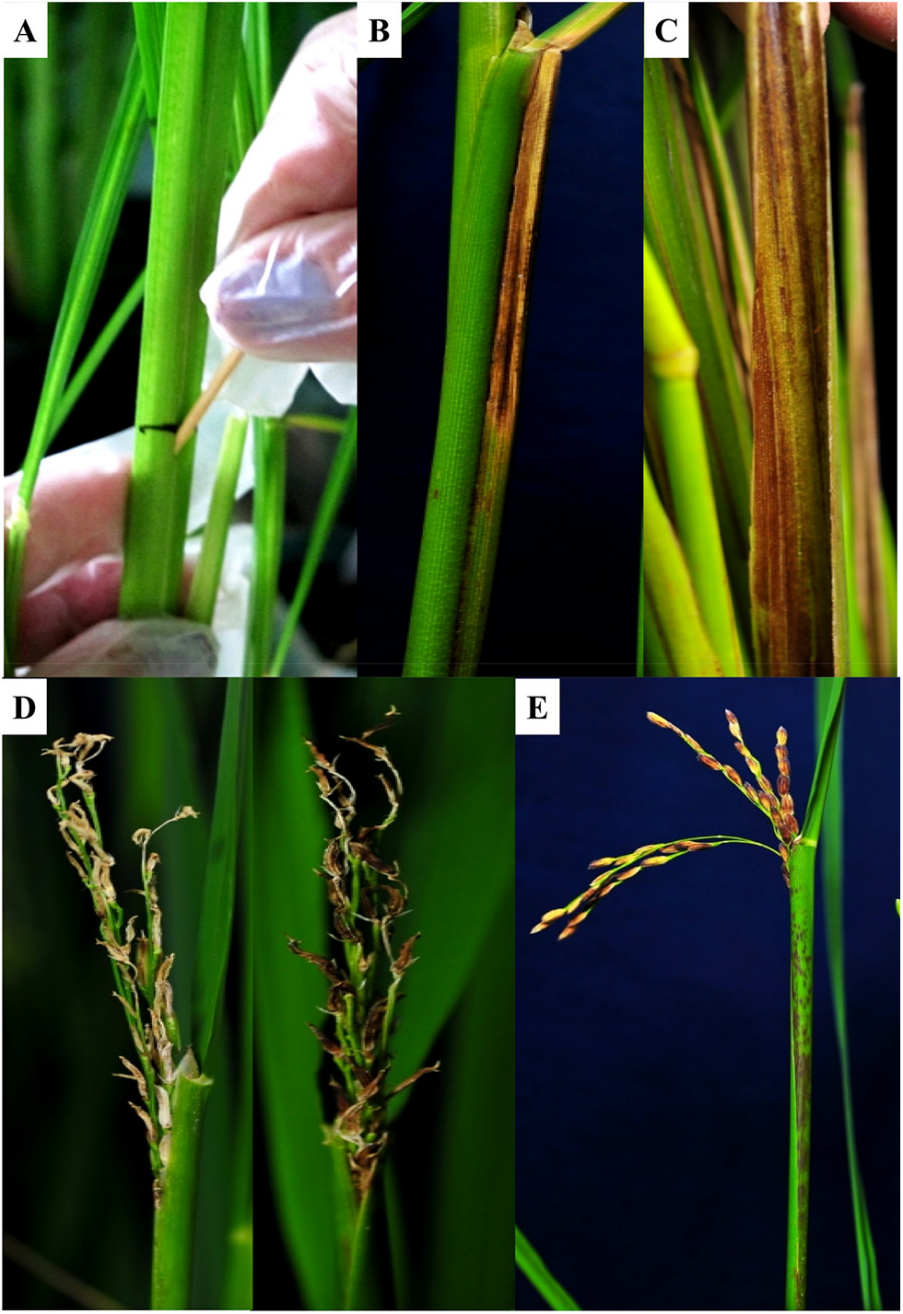
Infection caused by *P*. *fuscovaginae*-like strain IRRI 7007 in *O*. *sativa* cv. Azucena. **A)** Plants were inoculated at 45 days after transplanting using toothpick method. **B)** Symptom development along the sheath showing brown necrotic lesions. **C)** Discolored inner sheath. **D)** Poorly emerged panicles with brown to dark brown grains. **E)** Emerged panicles with discolored grains and progressive necrotic stripes at maturity stage.

To reproduce sheath brown rot and grain discoloration symptoms produced by *Pfv*-like pathogens, we inoculated the rice cultivars Azucena and Moroberekan using the toothpick method (Fig 1A). Phenotypic symptoms caused by the three *Pfv*-like strains were similar to *Pfv* as reported in Asia, Africa, and South America [6, 7, 16] which include the appearance of brown to reddish brown discolorations extending to the entire length of the sheath to its inner tissues (Fig 1B and 1C). Similar to the findings made by Cottyn *et al*. [22], *Pfv*-like strains were pathogenic on rice and showed variation in virulence spectrum. In order to validate pathogen spread, plants were kept until maturity to assess phenotypic symptoms in the grain. In most cases, panicles that emerged from the inoculated plants were necrotic and produced discolored grains that are often sterile compared with the control plants (Fig 1D and 1E). Fluorescent *Pseudomonas* colonies similar to *Pfv* were re-isolated from the infected sheath (data not shown). In addition to previous findings [22], these observations indicate that *Pfv*-like populations represented by IRRI 6609, IRRI 7007, and S-E1 harbor similar capabilities as that of *Pfv* to infect the rice host. Although sheath brown rot and seed discoloration phenotype assessment was not the aim of this study, we found that all strains were able to spread successfully across different tissues. Therefore, we decided to investigate the composition of *Pfv*-like factors contributing to bacterial sheath brown rot at the genomic level.

### Genome sequence of *Pfv*-like IRRI 6609 and IRRI 7007 and other available *Pfv* draft genomes

To date, the draft genomes of five *Pfv* and one *Pfv*-like strains are available in the public domain [33–35], representing a useful resource to consolidate our understanding of this novel pathogen population. To gain insight into the overall genome structure of *Pfv*-like, we generated high quality draft genomes of IRRI 6609 and IRRI 7007. Genome statistics of the strains IRRI 6609, IRRI 7007, S-E1 (Philippines), UPB0736 (Madagascar), CB98818 (China), ICMP 5940 (Japan), and DAR 77795 and DAR 77800 (Australia) are summarized in Table 1. Among all *Pfv*-like genomes, the assembly of IRRI 6609 produced the largest genome and the least number of scaffolds. Based on this, we considered IRRI 6609 as our reference genome for further analysis. It is not clear whether the difference in contig number between IRRI 6609 and IRRI 7007 is due to either the intrinsic differences between the strains or unresolved problems with the assembly. However, there is no reason to believe that contamination or low-quality reads are responsible for this difference. Interestingly, draft genomes of *Pfv* obtained from different sources [33–35] also show variability regarding genome size and number of protein coding genes (Table 1), suggesting that genome structure among groups may be more complex than we previously thought. Although not significant, we detected variation of G+C content between *Pfv* and *Pfv*-like genomes, with the *Pfv* genomes having an average of 61.31% while *Pfv*-like genomes had 63.12%. The G+C content has a phylogenetic signal over a short evolutionary time period [62], thus suggesting a close relatedness.

**Table 1:** General features of the eight rice-infecting *Pseudomonas* draft genomes.

	*Pseudomonas fuscovaginae*-like			*Pseudomonas fuscovaginae*					
	IRRI 6609	IRRI 7007	S-E1	CB98818	UPB0736	ICMP 5940		DAR 77795	DAR 77800
**Origin** ^a^	Philip	Philip	Philip	China	Madag		Aus	Aus	Aus
**No. contigs**	79	617	692	263	102		459	482	791
**N50 (kbp)**	355.2	27.0	92.3	44.5	205.3		47.4	39.6	17.8
**Largest contig size (kbp)**	746.8	133.1	305.2	202.4	605.8		189.6	142.3	96.1
**Total size (Mbp)**	7.14	6.73	6.55	6.54	6.35		6.37	6.25	5.97
**G+C content (%)**	63.25	63.00	63.13	61.4	61.46		61.2	61.40	61.10
**RNA coding genes**	133	127	113	140	125		56	53	51
**Protein coding genes**	6,342	6,699	5,897	6,433	5,689		6,367	6,035	7,202

^a^ Country names: Philip = Philippines, Madag = Madagascar, and Aus = Australia.

### *Pfv*-like strains are closely related to *Pfv*

**Fig 2:**
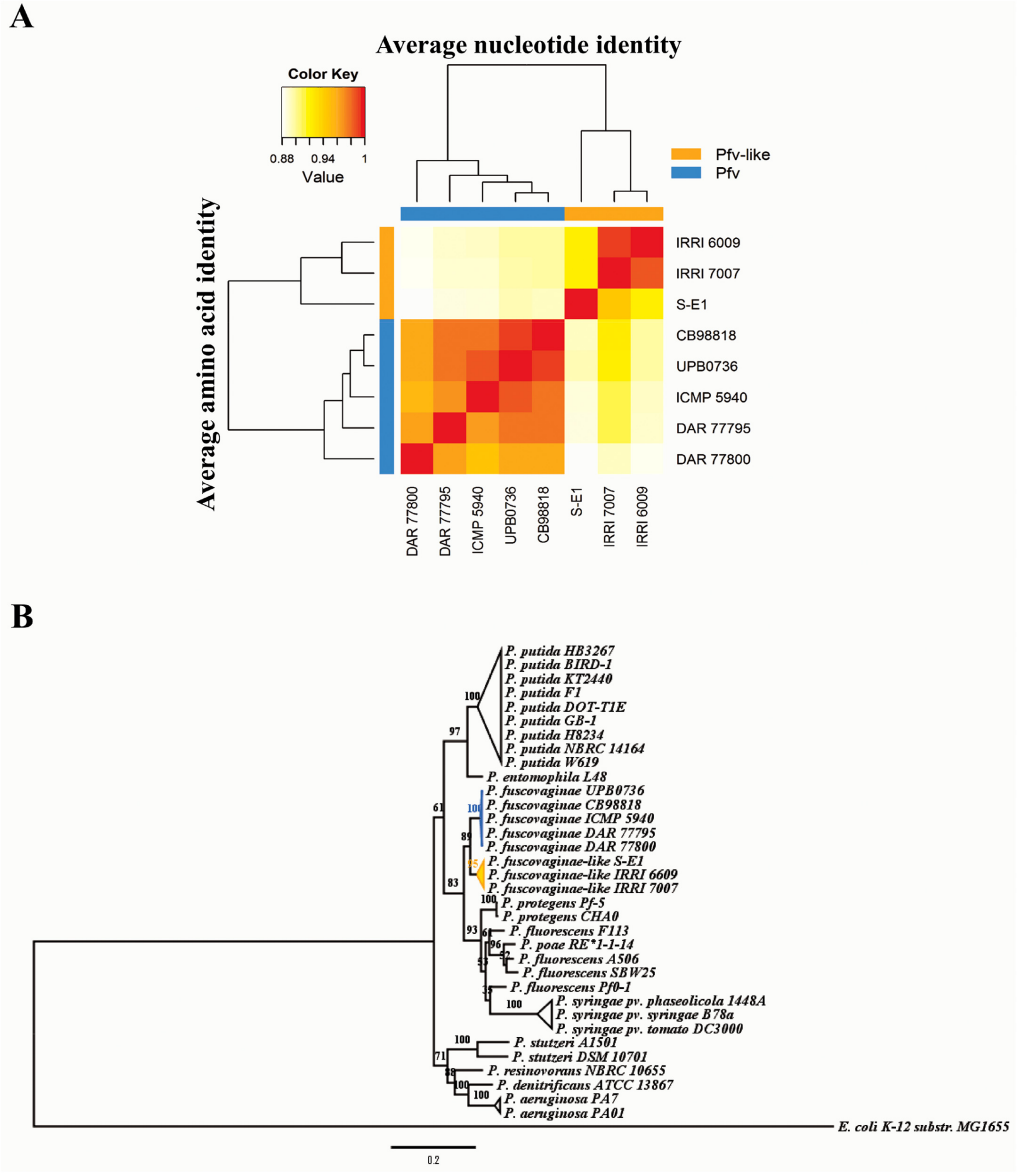
*P*. *fuscovaginae-like* (*Pfv*-like) strains are closely related to *P*. *fuscovaginae* (*Pfv*). **A)** Average nucleotide identity (ANI) and average amino acid identity (AAI) clustering analysis of the eight rice-infecting *Pseudomonas* draft genomes. Clustering analysis identified two separated groups involving *Pfv*-like strains (orange) collected in the Philippines and *Pfv* strains (blue) collected elsewhere. Values scale is depicted in red, orange, yellow, and white colors in ANI (horizontal) and AAI (vertical) pairwise comparison. Value cut-offs with >95% reflect the possibility of same species grouping. The heatmap was generated in the R package gplots using the heatmap.2 function. **B)** Phylogenetic reconstruction of rice-infecting *Pseudomonas* and closely related *Pseudomonas* species using the concatenated housekeeping *rpoB* and *rpoD*. Maximum likelihood was used to infer the phylogenetic relationship with bootstrap of 1000 using the RAxML software [61]. *Pfv* and *Pfv*-like are highlighted in blue and orange, respectively.

To get insight into the overall homology levels and to evaluate genetic relatedness among *Pfv* and *Pfv*-like genomes, we calculated both ANI and AAI values for the eight genomes [44, 63]. We found levels of homology that were inconsistent with a single monophyletic group. In our analysis, the ANI values ranged from 87.95% to 98.65% while the AAI values varied from 87.95% to 99.35% (Fig 2A). We built a distance matrix based on pair-wise ANI-AAI values which identified at least two subgroups within rice-infecting *Pseudomonas* (Fig 2A). The dendrogram topology clustered the *Pfv* strains from Madagascar, Japan, China, and Australia separately from those *Pfv*-like strains from the Philippines. Similar to previous reports [22], IRRI 6609, IRRI7007, and S-E1 appear to be more related to each other. Values that separate *Pfv* and *Pfv*-like groups were below the species boundary cut-off of 95% (Fig 2A). This result also correlates with the TETRA analysis that had a species delineation cut-off of ≥ 99% (S1 Fig). Using a one-way comparison approach, we also explored the distribution of homology between IRRI 6609 and 78 closely related *Pseudomonas* genomes (S1 Table). Results were more consistent with a continuous distribution rather than a clearly defined cluster, with average ANI values ranging from 82.57% for *P*. *stutzeri* (n = 6) genomes to 96.13% for *Pfv-*like (n = 2) genomes (S2 Fig). The data suggest that *Pfv*-like genomes were more related to *Pfv* than to any of the other 71 closely related *Pseudomonas* genomes. We also characterized genome ancestry using a phylogenetic analysis involving 34 *Pseudomonas* accessions. Using *rpoB* and *rpoD*, we found two separated clusters (Fig 2B) that correlated with the homology status of ANI and AAI, in which *Pfv* and *Pfv*-like populations share a common ancestor. To further support the phylogenetic relationship of *Pfv* and *Pfv*-like strains (Fig 2B), we constructed a robust evolutionary tree using 10 housekeeping genes and observed similar results (S3 Fig). It is clear that species definition in the genus *Pseudomonas* can be particularly problematic for some of the groups due to the intrinsic diversity in their genomic content [17, 21]. For instance, the *P*. *fluorescens* group actually involved multiple species [20, 21] occupying quite diverse ecological niches. Based on the whole genome comparison or phylogenetic inference, we did not find any evidence suggesting that *Pfv*-like strains may be considered as *Pfv sensu stricto*. Moreover, *Pfv* may be part of a species complex composed of different groups including the *Pfv*-like organisms analyzed in this study. Whether these strains can be considered a novel species or not will need further research efforts.

### *Pfv*-like and *Pfv* harbor high levels of structural polymorphism

**Fig 3:**
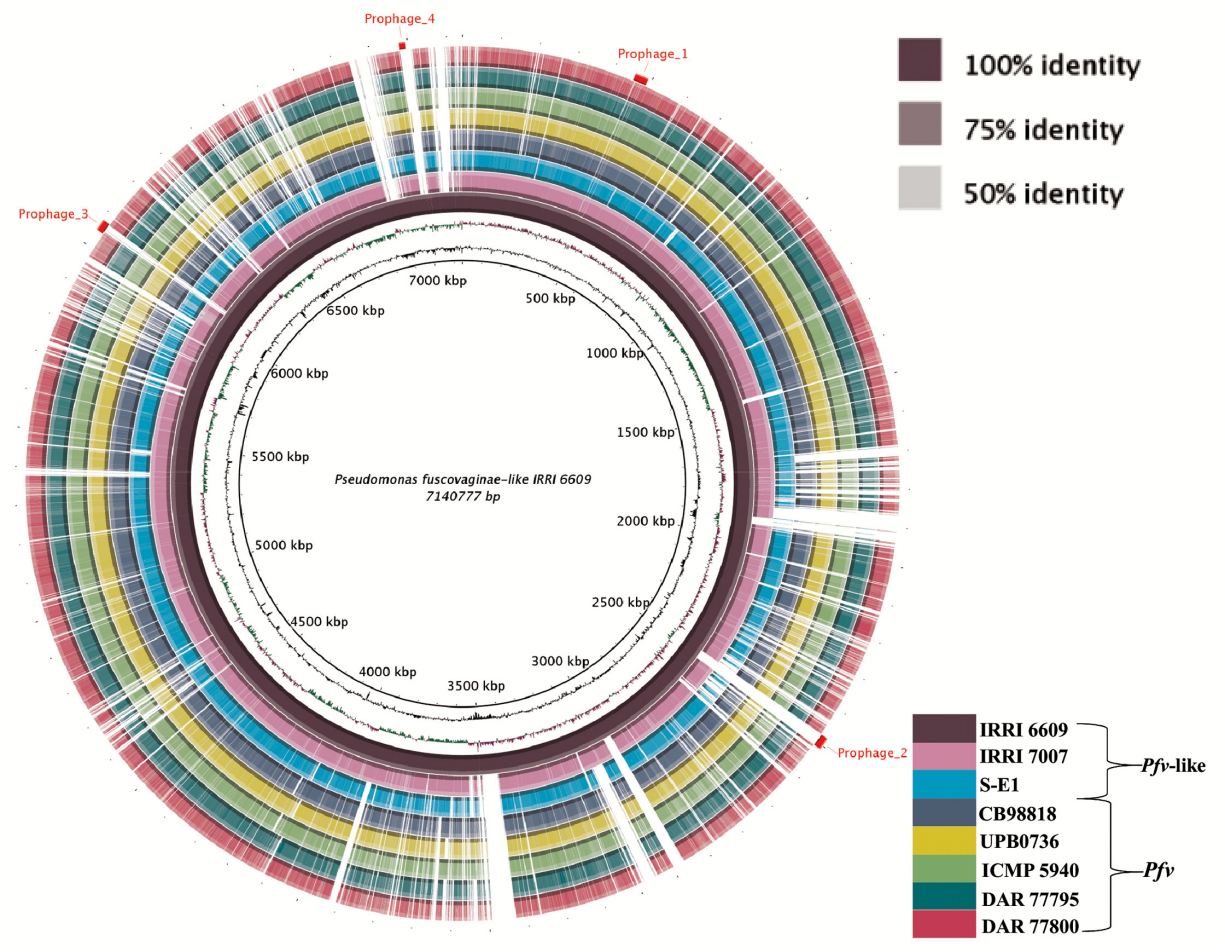
The genome of rice-infecting *Pseudomonas* harbor high level of structural polymorphism. Global comparison of eight rice-infecting *Pseudomonas* draft genomes using BLASTn. The inner most ring corresponds to the genomic position at IRRI 6609. The second and third rings indicate G+C content and G+C skew, respectively. The rest of the rings indicate presence and absence portions of the eight rice-infecting *Pseudomonas* draft genomes against IRRI 6609. Solid colors represent genomic regions with hits while white spaced represent gaps. *P*. *fuscovaginae* (*Pfv*) and *P*. *fuscovaginae*-like (*Pfv*-like) strains are depicted. Sequence identity is related to color intensity. Also included are locations of four intact prophage insertions found in *Pfv*-like IRRI 6609 (S4 Fig). The global alignment was visualized using BRIG [43].

A significant variation in the genomic composition of *Pseudomonas* groups has been reported recently [20, 64] and previous studies on *Pfv* have also found important genetic and biochemical variations among strains from different parts of the world [10, 35]. To understand the genome structure of rice-infecting *Pseudomonas* pathogens, we performed a comparative genomics analysis on eight draft genome datasets (Table 1). Whole genome alignments showed a high level of polymorphism among strains of *Pfv* and *Pfv*-like (Fig 3). Many syntenic blocks were interrupted by insertions, deletions, and rearrangements. A closer comparison of the eight genomes in terms of percentage of nucleotide identity and orthologous genes can be found in Table 2. Although *Pfv* and *Pfv*-like genomes appear to have many syntenic regions (Fig 3), our structural and gene content assessments illustrate a large degree of genomic diversity in *Pfv*-like strains. Loper *et al*. [20] also found a similar pattern when they analyzed members of the *P*. *fluorescens* subgroup that are closely related or belonged to the same taxa.

**Table 2:** Nucleotide identity and percentage of orthologous genes obtained in rice-infecting *Pseudomonas* draft genomes compared to IRRI 6609

Species	Strain	Nucleotide Identity (%)^a^	Percentage of orthologous genes
*Pseudomonas fuscovaginae*-like	IRRI 6609	100	100
*Pseudomonas fuscovaginae*-like	IRRI 7007	91.93	90.19
*Pseudomonas fuscovaginae*-like	S-E1	86.39	80.19
*Pseudomonas fuscovaginae*	CB98818	84.03	74.17
*Pseudomonas fuscovaginae*	UPB7036	84.21	76.22
*Pseudomonas fuscovaginae*	DAR 77795	83.89	71.13
*Pseudomonas fuscovaginae*	DAR 77800	84.52	60.56
*Pseudomonas fuscovaginae*	ICMP 5940	84.04	71.44

^a^ Identity based on BLASTn results

A considerable large proportion of prophage-related gene clusters were found in *Pfv* and *Pfv*-like strains (S4 Fig), indicating that they might have played an important evolutionary role in lateral gene transfer and contributed as well in the internal rearrangement of the genomes [65]. Out of the nine putative intact prophage clusters, three were present in more than one genome and five were found to be strain-specific. An overall assessment of prophage sequences in the eight genomes identified 644 phage-related genes, 37 novel non-phage related intact coding sequences, and 10 putative secreted proteins (S4 Fig). We showed that structural variation is consistent with major events of insertion/deletion in *Pfv*-like. While prophage insertions may not be the only mechanism incorporating foreign genes in the *Pfv* genetic pool, it is certainly contributing to genetic diversity. The presence of predicted secreted proteins within the inserted fragments may add an additional layer of functional adaptation as observed in other *Pseudomonas* groups [21].

### Pan-genome of *Pfv* and *Pfv*-like are highly accessorized

**Fig 4:**
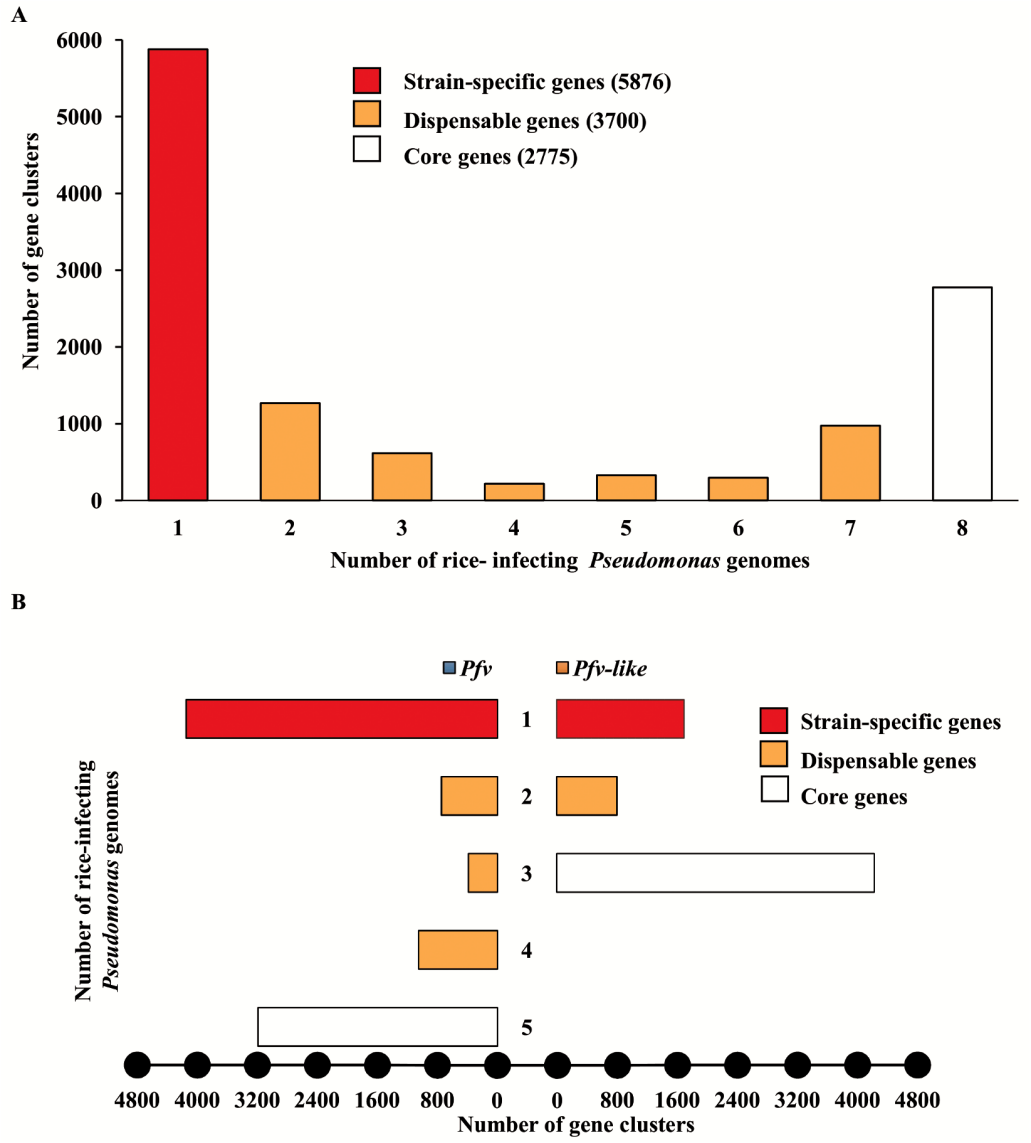
The pan-genome of rice-infecting *Pseudomonas* reveals high proportion of strain-specific genes. **A)** Distribution of the 12,351 orthologous gene clusters according to strain-specific genes (only in one genome = 1), dispensable genes (in more than one genome = 2 ≥ x ≤ 7), and core genes (in all genomes = 8). **B**) Orthologous gene distribution in the *P*. *fuscovaginae* (blue) and *P*. *fuscovaginae*-like (orange) genomes depicting number of core, dispensable, and strain-specific gene clusters.

The pan-genome is the overall repository of genes within a species which is further subdivided into core genes shared by all members, dispensable genes shared by more than one member, and strain-specific genes which can be found only in one member [66]. To get insight into the pan-genome of rice-infecting *Pseudomonas*, we first grouped all 50,664 predicted open reading frames from the eight genomes into 12,351 orthologous gene clusters. Interestingly, the majority of clusters were present in only one of the strains, suggesting that *Pfv* and *Pfv*-like have highly accessorized pan-genomes (Fig 4A). It is not likely that the reduced core genome observed in *Pfv* and *Pfv*-like is due to poorly assembled draft genomes. All strains showed big genome sizes, high number of coding genes, and random distribution of strain-specific genes located in syntenic regions. For that reason, we predicted that fully assembled *Pfv* and *Pfv*-like genomes will produce a slight increase in the core genome but will not change drastically the pattern of strain-specific genes.

To explore the genetic differences within rice-infecting *Pseudomonas* populations in more detail, we repeated the analysis using *Pfv* and *Pfv*-like genomes independently and found that 77% of the 12,351 orthologous clusters were present in *Pfv*. As expected, the number of strain-specific clusters remained high, which suggests that each strain maintains its own repertoire of genes (Fig 4B). Although *Pfv*-like have only three strains and share the 54% of the overall orthologous clusters, we identified 1,696 strain-specific clusters in this dataset (Fig 4B). In line with these findings, the functional annotation of *Pfv* and *Pfv*-like core genes was also consistent with metabolic versatility (S5 Fig). We observed an enrichment in core genes related to transcription, inorganic ion transport metabolism, and intracellular trafficking between *Pfv*-like and *Pfv*. The examined trend suggests that each *Pfv*-like and *Pfv* genome is extremely plastic and harbored a battery of genes potentially involved in their adaptation to a number of hosts or different environments. As of the moment, we cannot discard that sampling bias may also contribute to the observed differences.

Accurate estimation of bacterial pan-genome can be highly dependent on the number and diversity of strains involved in the analysis [67]. To estimate if *Pfv* has an open or close pan-genome, we used a power law regression model [68] to establish the relationship between cluster size and strain number. Our result was consistent with an infinite or open *Pfv* pan-genome (S6 Fig), in which the number of genes in the pool increased exponentially with each genome added without reaching a clear plateau. The number of core genes was also constantly decreasing with the addition of each new genome (S6 Fig). The inferred pan-genome also correlates with high levels of structural variation (Fig 3), low levels of sequence homology as measured by ANI-AAI analysis (Fig 2A), and high proportion of strain-specific genes (Fig 4A and 4B). Therefore, our findings are not surprising since *Pfv* appears to be an opportunistic pathogen with a broad host range and mechanism that allow frequent gene exchange in multiple niches. It is likely that *Pfv*-like groups are also capable to use multiple sources and colonize different environments. A similar situation was described for other *Pseudomonas* groups that have evolved high levels of genomic plasticity such as *P*. *fluorescens* and *P*. *syringae* pathovars [19, 21, 64]. Despite the limited understanding about *Pfv* and *Pfv*-like life cycle, the analysis of the pan-genome structure is giving us important clues into the biology of these rice pathogens.

### *Pfv*-like has four major secretion apparatus

**Fig 5:**
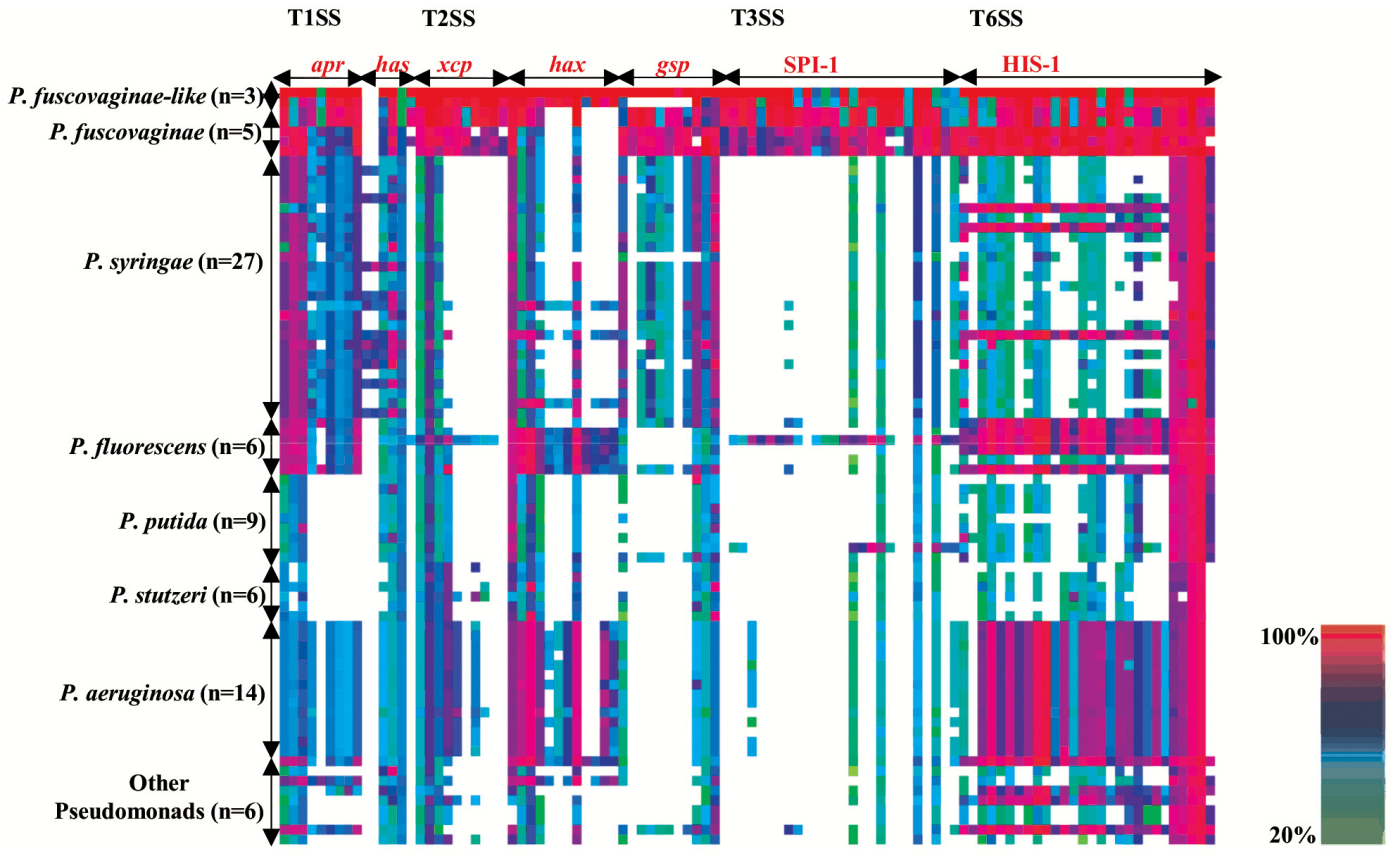
Comparative genomic analysis of rice-infecting *Pseudomonas* secretion apparatus. Genetic components of T1SS, T2SS, T3SS and T6SS apparatus of *P*. *fuscovaginae-like* (*Pfv*-like) IRRI 6609 was used to compare against 79 closely related *Pseudomonas* genomes (S1 Table). The *apr*, *has*, *xcp*, *hxc*, *gsp*, SPI-1, and HSI-1 are previously characterized gene clusters found within each secretion system. Horizontal axis describes the number of species used for comparison. The rows were sorted by amino acid sequence identity with threshold set at 20%. The heat map was visualized in CodaChrome. Homology range values are shown in bottom right.

Plant pathogenic bacteria use a combination of secretion systems to modify the surrounding environment and to interact with hosts and other microbes [69]. Using homology pairwise comparison, we investigated the composition and conservation of the secretion apparatus in *Pfv*-like genomes and found intact Type I (T1SS), Type II (T2SS), Type III (T3SS), and Type VI (T6SS) systems (Fig 5 and S4 Table). All these secretion systems are commonly found in host-associated bacteria and have been reported in *Pfv* draft genomes [33–35].

We then used a comparative approach to investigate the level of conservation of the *Pfv*-like secretion apparatus in 79 closely related *Pseudomonas* genomes, including rice-infecting *Pseudomonas*. The T1SS, which includes the *apr* and *has* clusters [70], was highly conserved across all *Pseudomonas* genomes with the exception of *P*. *putida* and *P*. *stutzeri* (Fig 5). This cluster includes an alkaline protease which has a strong homology to *arpA* [71] and additional genes with predicted exoprotease activities. We found three different T2SS gene clusters in *Pfv*-like. The *xcp* cluster showed strong homology with *P*. *aeruginosa* [70] while the *gsp* cluster appears to be conserved within *P*. *syringae* genomes [72]. Interestingly, the *hxc* cluster is only present in *Pfv*-like strains and has homology to *P*. *fluorescens* and *P*. *aeruginosa* genomes (Fig 5). Substrates for T2SS, such as CWDE, were also identified in *Pfv*-like genomes (see below).

Interestingly, *Pfv* and *Pfv*-like populations lack the typical *hrp/hrc* T3SS found in other plant pathogenic *Pseudomonas* but carry another T3SS family member, the SPI-1 (Salmonella Pathogenicity Island 1) (Fig 5 and S4 Table). Loss-of-function experiments suggest that *Salmonella enterica* SPI-1 contributes to virulence by secreting type III effectors during human cell colonization [73]. Recent reports that showed SPI-1 in several plant-associated bacteria [21, 33, 74–77] suggested alternative functions outside the mammalian system [78]. Indirect evidence from *Arabidopsis thaliana* and *Nicotiana tabacum* showed that *S*. *typhimurium* SPI-1 mutants were unable to suppress plant immune response [79, 80]. In our analysis, SPI-1 was poorly conserved in almost 70 *Pseudomonas* genomes. Only the *P*. *fluorescens* strain F113, a plant growth-promoting bacterium, carries an intact SPI-1 cluster [21]. Interestingly, our analysis identified only a few candidate type III effectors in the IRRI 6609 and IRRI 7007 genomes but their role during host colonization is still unclear. Whether SPI-1 is used by *Pfv* or *Pfv*-like to deliver unknown type III effectors to interact with rice or with alternative hosts outside the plant kingdom remains to be an area that should be investigated further.

T6SS showed strong homology to the corresponding *hcp* Secretion Island 1 (HSI-1) [81] encoded in the genome of all 14 *P*. *aeruginosa*, 5 out of 6 *P*. *fluorescens*, and 3 out of 27 *P*. *syringae* strains (Fig 5). This finding is not surprising since T6SS is highly conserved among pathogenic bacteria and has been implicated in multiple functions. For instance, *P*. *aeruginosa* uses T6SS to secret the effector protein Tse2 as a component of a toxin-substrate system that mediates interactions between bacteria [82]. Meanwhile, T6SS mutants of the seed-borne pathogen *Acidovorax citrulli* were impaired in seed-to-seedling transmission in citrus plants [83]. More recently, a *Pfv* Tn5 mutant of the T6SS showed impaired colonization of rice tissue [32], suggesting that T6SS contributes to pathogenicity. Even though functional data that links *Pfv*-like secretion system with virulence in rice is still missing, it is likely that each secretion system was acquired independently. This observation indicates a diversity of functional roles that is aligned with the general idea that *Pfv*-like, similar to *Pfv*, is capable of colonizing multiple environments.

### The core secreted repertoire of *Pfv* and *Pfv*-like has signatures of positive selection

**Fig 6:**
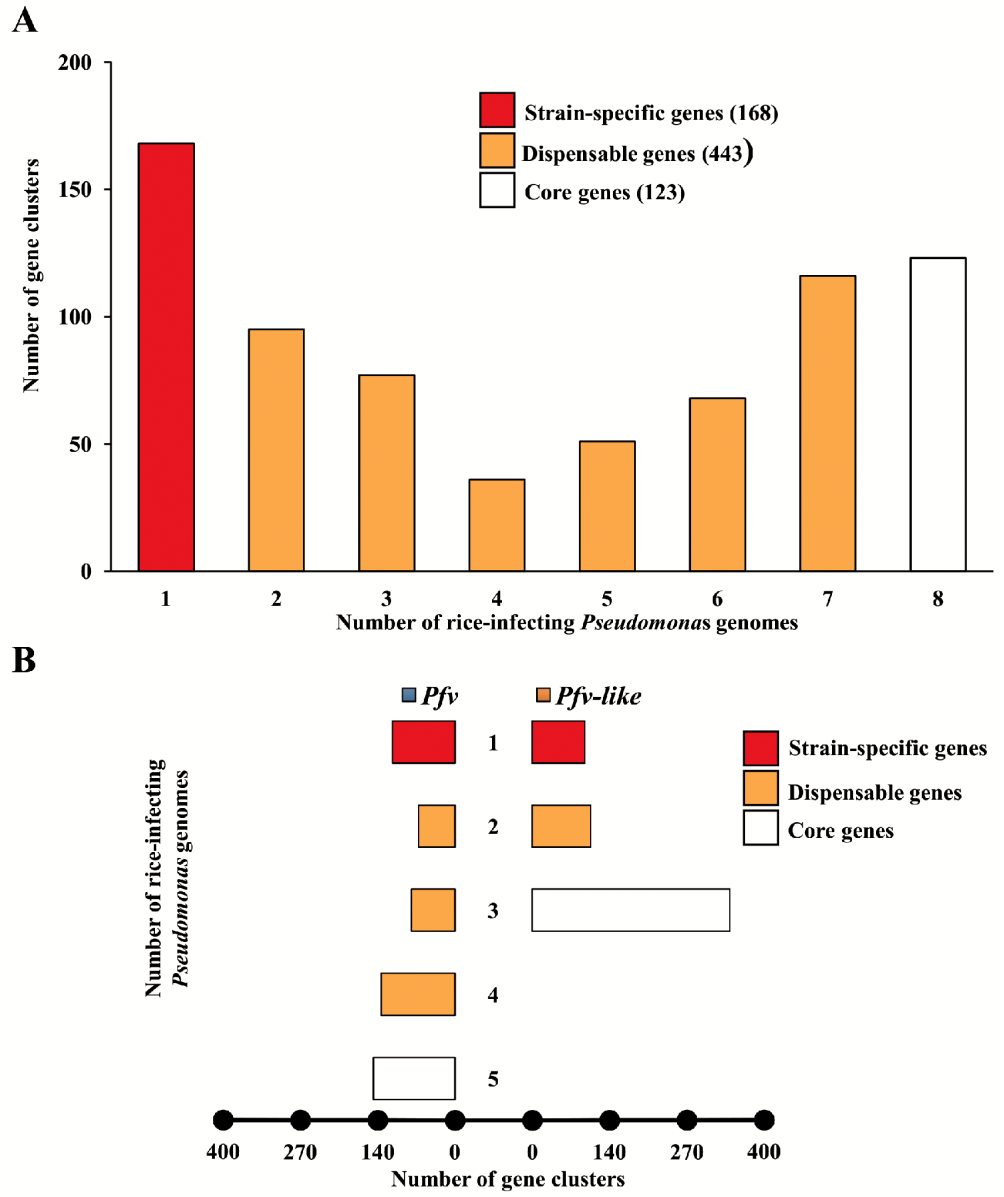
The secretome of rice-infecting *Pseudomonas* has high proportion of dispensable genes. **A)** Distribution of the 729 orthologous gene clusters in the secretome according to strain-specific genes (only in one genome = 1), dispensable genes (in more than one genome = 2 ≥ x ≤ 7) and core genes (in all genomes = 8). **B**) Orthologous gene distribution in the *P*. *fuscovaginae* (blue) and *P*. *fuscovaginae*-like (orange) genomes depicting number of core, dispensable, and strain-specific gene clusters.

Similar to other bacterial pathogens, rice-infecting *Pseudomonas* are predicted to secrete a number of effector proteins that contribute to disease progression. To investigate the secretion capabilities in *Pfv* and *Pfv*-like genomes and to identify putative core and dispensable secretome, we used a combination of prediction tools. We first used SignalP [50] and identified 4,244 proteins that had canonical secretion signals in all the eight genomes. Then, we removed 715 proteins which were predicted to have at least one transmembrane domain, as those may represent membrane-anchored proteins. Using the same approach as described above, we estimated 734 orthologous gene clusters as the overall repertoire of putative secreted proteins in the eight genomes (Fig 6A). Among those, 168 corresponded to strain-specific genes (Fig 6A). We also analyzed the distribution of putative secreted proteins in *Pfv* and *Pfv*-like groups independently. Both groups maintained high proportion of unique genes reaching 20.98% for *Pfv* and 17.05% for *Pfv*-like (Fig 6B). These findings point out to a unique set of secreted proteins in each rice-infecting *Pseudomonas* genome consistent with multiple functional capabilities. In the same way, Baltrus *et al*. [64] found dramatic variation in the number and distribution of effector genes across a range of *P*. *syringae* clades from different hosts. Recent analysis of the *P*. *fluorescens* F113 strain harbored an unprecedented combination of unique genes related to rhizosphere colonization [21]. Therefore, it can be assumed that some *Pseudomonas* groups probably evolved to maximize the dispensable secretome as a strategy to occupy a range of environments.

**Fig 7:**
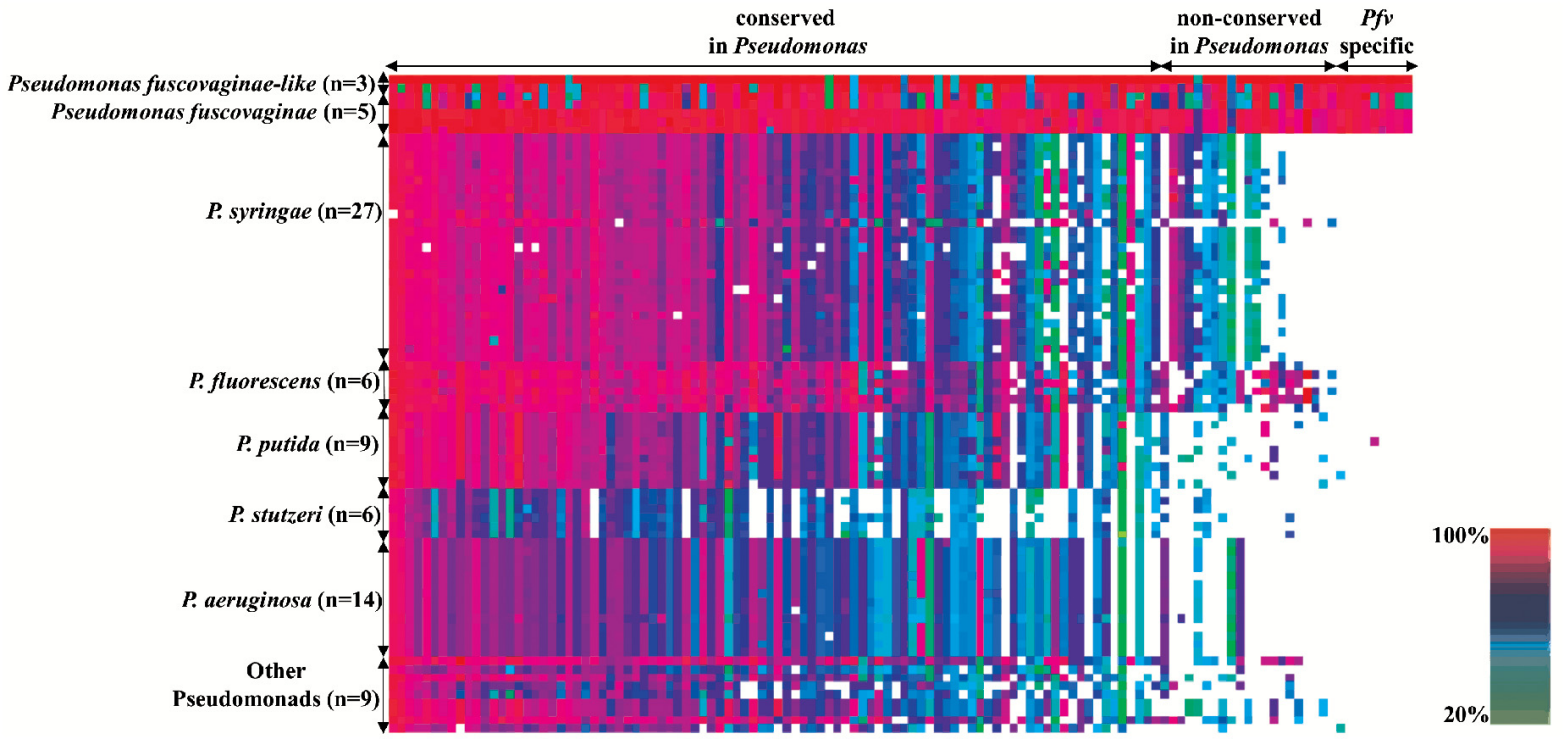
The core secretome of rice-infecting *Pseudomonas* harbor unique genes. Conservation of core secreted proteins from rice-infecting *Pseudomonas* was evaluated in 79 closely related *Pseudomonas* genomes (S1 Table). Columns were sorted by averaging the amino acid identity to identify conserved and species-specific proteins using threshold of 20%. Secreted proteins are also classified in: conserved in all *Pseudomonas*, non-conserved in all *Pseudomonas*, and *Pfv-* and *Pfv*-like-specific. Horizontal axis describes the number of species used for comparison. The heat map was visualized in CodaChrome [40]. Homology range values are shown in bottom right.

Importantly, we identified 123 core genes that codified for putative secreted proteins in all the rice-infecting *Pseudomonas* genomes (Fig 6A and 6B, and S5 Table). Most of the candidate core secretome was associated with transport, catalytic, and binding activities (S6 Table), which are the possible molecular functions of the core secreted proteins. To further categorize the level of conservation in other *Pseudomonas* genomes, we used homology comparison and identified 31 candidate genes that were homologous to known plant pathogenic *Pseudomonas* (Fig 7). Among these, 13 putative secreted proteins were unique to rice-infecting *Pseudomonas* strains. Since all strains were isolated from rice and showed exactly the same brown rot and seed discoloration symptoms, this set of genes is likely to play a role during interaction with the host plant. The other 96 secreted proteins were conserved among free living and pathogenic *Pseudomonas* isolated from a range of eukaryotic hosts (Fig 7). Future research is needed to understand the role of these secreted proteins during interaction with rice.

**Fig 8:**
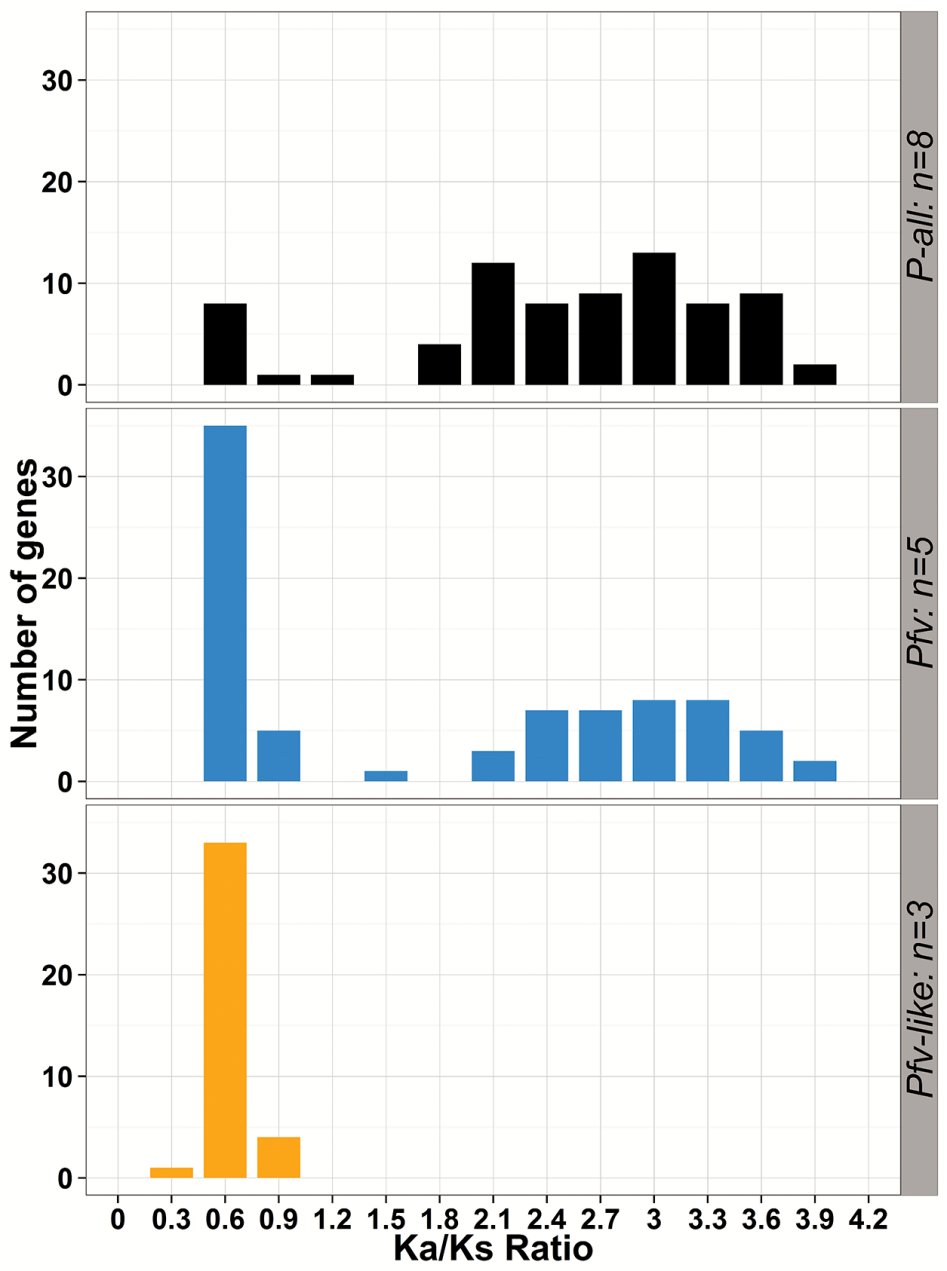
The core secretome of rice-infecting *Pseudomonas* has signatures of positive selection. Distribution of Ka/Ks ratio for 123 protein-coding genes, calculated with Yn00 [59] method on rice-infecting *Pseudomonas*-all (black, *P*-all, n = 8), *P*. *fuscovaginae* (blue, *Pfv*, n = 5), and *P*. *fuscovaginae*-like (orange, *Pfv*-like, n = 3) datasets. All secreted protein selected on this graph have *p*-values ≤ 0.01.

A common feature of effector genes from plant pathogenic microbes is the strong signature of positive selection [84–86]. To characterize the selection pressures underlying the *Pfv* and *Pfv*-like core secretome and to identify candidate effector genes, we calculated Ka*/*Ks ratio using Yn00 [59] on alignments of 123 orthologous loci across the eight genomes. Using a cutoff p-value of 95%, we found that Ka value was greater than Ks (ω = Ka/Ks > 1) in 75 of 123 genes (Fig 8). We obtained ω values ranging from 0.45 to 4.0 (average of 2.4). When we analyzed each group separately, *Pfv* secretome ω values ranged from 0.37 to 3.76 (average of 1.65) while *Pfv*-like ranged from 0.14 to 0.8 (average of 0.48) (Fig 8). Among the 75 selected genes, we found high ω values in 3 out of 13 genes that were specific to rice-infecting *Pseudomonas* (Fig 8). This result shows significant differences in the number of genes under selection between *Pfv* and *Pfv*-like. Whether this difference is due to variation in selection pressure or sampling bias between groups is still unknown. Collectively, our findings point out that natural selection is continuously shaping the secreted repertoire of the rice-infecting *Pseudomonas*, which is a common pattern for other *Pseudomonas* pathogens [85, 87].

### Additional features potentially involved in pathogenicity of *Pfv*-like

To further explore the presence of additional pathogenicity mechanisms within the *Pfv*-like genome, we identified putative genes involved in secondary metabolism biosynthesis, hormone metabolism, motility, cell adhesion, pilus formation, and cell wall degradation (S3 and S4 Tables). All of the probable secondary metabolite gene clusters of *Pfv*-like are found in S4 Table. Three gene clusters related to AHL biosynthesis and quorum sensing were identified. *Pfv* harbors both AHL clusters (PfsI/R and PfvI/R) described in Mattiuzzo *et al*. [28] and one additional putative novel cluster yet to be characterized (S3 and S4 Tables). Additionally, two classes of siderophores biosynthetic genes were located in the genomes of *Pfv*-like. The cluster for pyoverdine biosynthesis found in *Pfv*-like is homologous to the *pvd* gene cluster from *P*. *aeruginosa* [88]. Pyoverdine gives *Pseudomonas* spp. their fluorescent pigments and is associated with iron acquisition [88]. We also found the *acs* gene cluster that is associated to achromobactin production [89]. Achromobactin, just like pyoverdine, appears to facilitate iron acquisition as an alternative function. Mutant strains of *P*. *syringae* pv. *phaseolicola* 1448a showed that pyoverdine and achromobactin were not essential in causing halo blight in beans [90]. In addition, the pyoverdine production of *P*. *fluorescens* F113 was effective in inhibiting the growth of *Pectobacterium atrosepticum in vitro* [91]. Whether the production of pyoverdine and achromobactin is important for microbial competition or during host pathogenicity of *Pfv*-like is still not known.

We found three non-ribosomal peptide (NRP) gene clusters involving at least 69 genes (S3 and S4 Tables). Two of these clusters showed no homology to any NRP cluster in other *Pseudomonas* species. Moreover, the biosynthetic products could not be identified. We also predicted a gene that codifies for tryptophan 2-monooxygenase, which is an important enzyme in auxin anabolism (S4 Table). Heterologous expression of this gene in *A*. *thaliana* promoted susceptibility to the bacterial pathogen *P*. *syringae* pv. *tomato* DC3000 [92]. *Pfv-*like genome also harbored T4 pili and flagella formation gene clusters which are important in bacterial adhesion to the cell and motility, respectively. In contrast to plant pathogens that cause rotting [93], *Pfv*-like appeared to have reduced repertoire of CWDE. From the 85 proteins predicted to have carbohydrate-active enzymes, only three harbored a canonical secretion signal (S4 Table). Some of the putative pathogenicity factors found in *Pfv*-like were also present in *Pfv*. The tryptophan 2-monooxygenase gene, clusters involved in pilus and flagella formation, biosynthetic clusters of achromobactin and pyoverdine, and the second putative NRP gene cluster were all conserved in *Pfv*, suggesting similar mechanisms. However, the third quorum sensing, and the two remaining NRP gene clusters appear to be unique to *Pfv*-like (S4 Table).

### *Pfv*-like induce multiple putative virulence factors during its interaction with rice

Infection of host plants by pathogenic bacteria is a complex process that requires the coordinated expression of many virulence components [94]. To determine the contribution of *Pfv*-like putative virulence factors during rice colonization, we analyzed the expression of 19 pathogen genes during a time course infection experiment using semi-quantitative RT-PCR. Total RNA was isolated from rice sheaths at 0, 3, 24, 48, and 72 hpi of strain IRRI 7007 in rice cv. Azucena. Selected genes are listed in S4 Table and represents: secretion apparatus T1SS (1), T2SS (3), T6SS (1), and SPI-1 (1); one gene involved in T4 pili formation; one flagella gene; two putative CWDEs; one gene predicted to be involved in putative NRP biosynthesis; six core secreted proteins; and two random core genes present in IRRI 6609 and IRRI 7007 (S3 and S4 Tables).

We detected transcript accumulation in 14 of 19 pathogen genes in at least one of the examined time points (S7 Fig). As expected, the pattern of expression varied considerably among genes. Some genes were constitutively expressed while others were induced as early as 3 hpi or down regulated after infiltration. Interestingly, genes that formed the secretion apparatus and those presumed to be secreted throughout this mechanism were detected at early stages of bacterial infection (S7 Fig). A putative alkaline proteases (PF66_01465), which is known to be secreted by T1SS, was induced at an early time point. In the same way, two genes with predicted CWDE activity (PF66_04809 and PF66_04639) were expressed during the first hours of infection. However, none of the genes representing the T2SS apparatus (PF66_01622, PF66_04896, and PF66_01114) were induced, suggesting that CWDE may be secreted throughout alternative pathways. Two putative methionine aminopeptidase with a predicted T3 secretion signal were expressed in this experiment (S7 Fig) The predicted T3SS effector gene PF66_01486 showed detectable levels at 3 hpi and was down regulated afterwards while PF66_00566 showed constitutive expression (S7 Fig). Recent experimental data showed that T6SS might be important for *Pfv* pathogenicity [32]. In our experiment, PF66_03771, which is part of T6SS was upregulated during the first three hours. These results may highlight the contribution of at least three secretion systems during the infection of *Pfv*-like in rice sheath.

The gene involved in membrane adhesion (PF66_01105) was found to be expressed at 3 hpi while the gene involved in locomotive function (PF66_05042) was highly expressed at all time points. Four (PF66_04809, PF66_01042, PF66_04639, and PF66_00996) out of six predicted secreted genes showed very early response during the interaction of rice and the *Pfv*-like strain IRRI 7007. The gene PF66_00996, which has a putative copper oxidase function, is also specific for *Pfv* and *Pfv*-like and has strong signature of positive selection (S7 Fig). Similar to known effector proteins, the induction of candidate core secreted genes during the early stage of infection may suggest the important functional role of PF66_04809, PF66_01042, PF66_04639, and PF66_00996 in *Pfv*-like pathogenicity. At a later stage (72 hpi), most of the tested genes had low level of expression. Overall, data obtained here revealed that *Pfv*-like interaction with rice sheath is accompanied by transcript accumulation of genes involved in secretion, adhesion, and trafficking, representing a concerted virulence mechanism to infect rice.

## Conclusion

In this study, we focused on understanding the complexity of rice-infecting *Pseudomonas* genomes that cause sheath brown rot and the underlying mechanisms that might govern its virulence in rice. Rather than genetically homogeneous lineages, *Pfv* and *Pfv*-like appear to have an open pan-genome with each isolate representing a very distinct lineage carrying its own repertoire of accessory genes. Structural variation may reflect major events of insertion-deletion in a genome that allows frequent gene exchange. The plasticity in genome content, the overall diversity of metabolic capabilities, and the functional adaptation of its secretome are all consistent with the idea that *Pfv* and *Pfv*-like are able to occupy multiple niches. Similar to other *Pseudomonas* species that show intrinsic genetic variation and continuous distribution of genetic diversity [17, 20, 21], *Pfv* and *Pfv*-like appear to represent different phylogenetic groups. However, the phylogenetic status of these strains needs to be analyzed in the context of *Pfv* meta-population. At the genomic level, *Pfv*-like harbors many distinct mechanisms that are potentially involved in rice colonization. Many of the genes involved in secretion, adhesion, and trafficking are activated during the early stages of infection, thereby highlighting its potential role during rice pathogenicity. We predicted a conserved set of secreted proteins in the pan-genome of *Pfv*-like and *Pfv*, most of which have strong signatures of positive selection. These observations reveal a unique evolutionary pathway of rice-infecting *Pseudomonas* in the agricultural landscape but also explain partially the ability of *Pfv* to infect a broad range of host plants outside the rice cropping system. Functional validation of core and strain-specific genes as well as an assessment of phenotypic differences between *Pfv* and *Pfv*-like groups will be important to reveal the conserved mechanism of infection and the unique set of metabolic capabilities of these populations. We hope these observations can lead to better strategies of control, monitoring and management of sheath blight rot in the rice plant.

## Supporting Information

S1 Fig Tetranucleotide frequency correlation coefficients (TETRA) of eight rice-infecting *Pseudomonas* genomes. Clustering analysis differentiates *P*. *fuscovaginae*-like (*Pfv-like*) collected in the Philippines and *P*. *fuscovaginae* (*Pfv*) collected elsewhere. Values scale is depicted in red, orange, yellow, and white colors in TETRA pairwise comparison. Value cut-offs with >99% reflect the possibility of same species grouping. The heatmap was generated in the R package gplots using the heatmap.2 function.
(TIFF)

S2 Fig Box plot representing average nucleotide identity (ANI) of *P*. *fuscovaginae*-like IRRI 6609 against 79 *Pseudomonas* genomes. The middle line in each box plot represents the mean of ANI values for a particular taxonomic group. Also shown in each box plot are the minimum and maximum values. The number of genomes is denoted in brackets. All *Pseudomonas* genomes used in this analysis are listed in S1 Table.
(TIFF)

S3 Fig Phylogenetic reconstruction of rice-infecting *Pseudomonas* and closely related species using the concatenated housekeeping genes: *acsA*, *aroE*, *dnaE*, *guaA*, *gyrB*, *mutL*, *ppsA*, *pyrC*, *recA*, and *rpoB*. Maximum likelihood was used to infer the phylogenetic relationship with bootstrap of 1000 using the RAxML software [61]. Groups *Pfv*-like and *Pfv* are highlighted as orange and blue, respectively.
(TIFF)

S4 Fig Composition of prophage insertions among eight rice-infecting *Pseudomonas* genomes. **A)** Distribution of intact prophage insertions are color-coded among rice-infecting *Pseudomonas* strains. Prophage ID is based on PHAST database [38]. All intact prophage have scores >90 [38]. **B)** Bar plot distribution of the number of genes found within all prophage regions in each of the eight rice-infecting *Pseudomonas* draft genomes. Non-phage related genes are further classified as secreted and non-secreted.
(TIFF)

S5 Fig Functional distribution of core genes from rice-infecting *Pseudomonas* using cluster of orthologous groups (COG) classification. Bar plots representing number of genes in each COG category per rice-infecting *Pseudomonas*-all (black, n = 8), *P*. *fuscovaginae* (blue, n = 5) and *P*. *fuscovaginae-like* (orange, n = 3).
(TIFF)

S6 Fig Estimation of pan-genome and core genome size of *P*. *fuscovaginae (Pfv)* based on information from five draft genomes using the equation described by Tettelin *et al*. [68]. Black dots represent standard error; X and Y axes represent genome size and number of genomes, respectively.
(TIFF)

S7 Fig Time course expression analysis of eighteen putative pathogenicity-related *P*.*fuscovaginae*-like genes during rice sheath colonization using semi-quantitative RT-PCR. Rice sheath (*O*. *sativa* cv. Azucena) infected with *P*.*fuscovaginae*-like strain IRRI 7007 was evaluated at 0, 3, 24, 48, and 72 hpi. Gen ID and functional classification are indicated in both sides.
(TIFF)

S1 Table List of *Pseudomonas* genomes used for comparative analysis in this study. The 79 *Pseudomonas* genomes are listed according to species, strains and their corresponding GenBank ID.
(DOCX)

S2 Table List of housekeeping genes used in the construction of phylogenetic tree. Locus ID and sequence correspond to *P*. *fuscovaginae*-like strain IRRI 6609.
(XLSX)

S3 Table Primer pairs used in the expression analysis of *P*. *fuscovaginae*-like IRRI 7007 genes during its interaction with rice. (XLSX)

S4 Table Putative pathogenicity related genes found in *P*. *fuscovaginae*-like genome. (DOCX)

S5 Table Functional annotation of 123 core secreted proteins identified among eight rice-infecting *Pseudomonas* genomes. Annotation was done using Pfam. Locus ID based on *P*.*fuscovaginae*-like IRRI 6609.
(XLSX)

S6 Table Predicted molecular function of the core secreted proteins of rice-infecting *Pseudomonas* using Gene ontology (GO) term. (DOCX)
