# Supplementary figures and images for: Rice-Infecting Pseudomonas Genomes Are Highly Accessorized and Harbor Multiple Putative Virulence Mechanisms to Cause Sheath Brown Rot
Source: PLoS One. 2015 Sep 30;10(9):e0139256. doi: 10.1371/journal.pone.0139256 (PMC4589537; doi:10.1371/journal.pone.0139256)

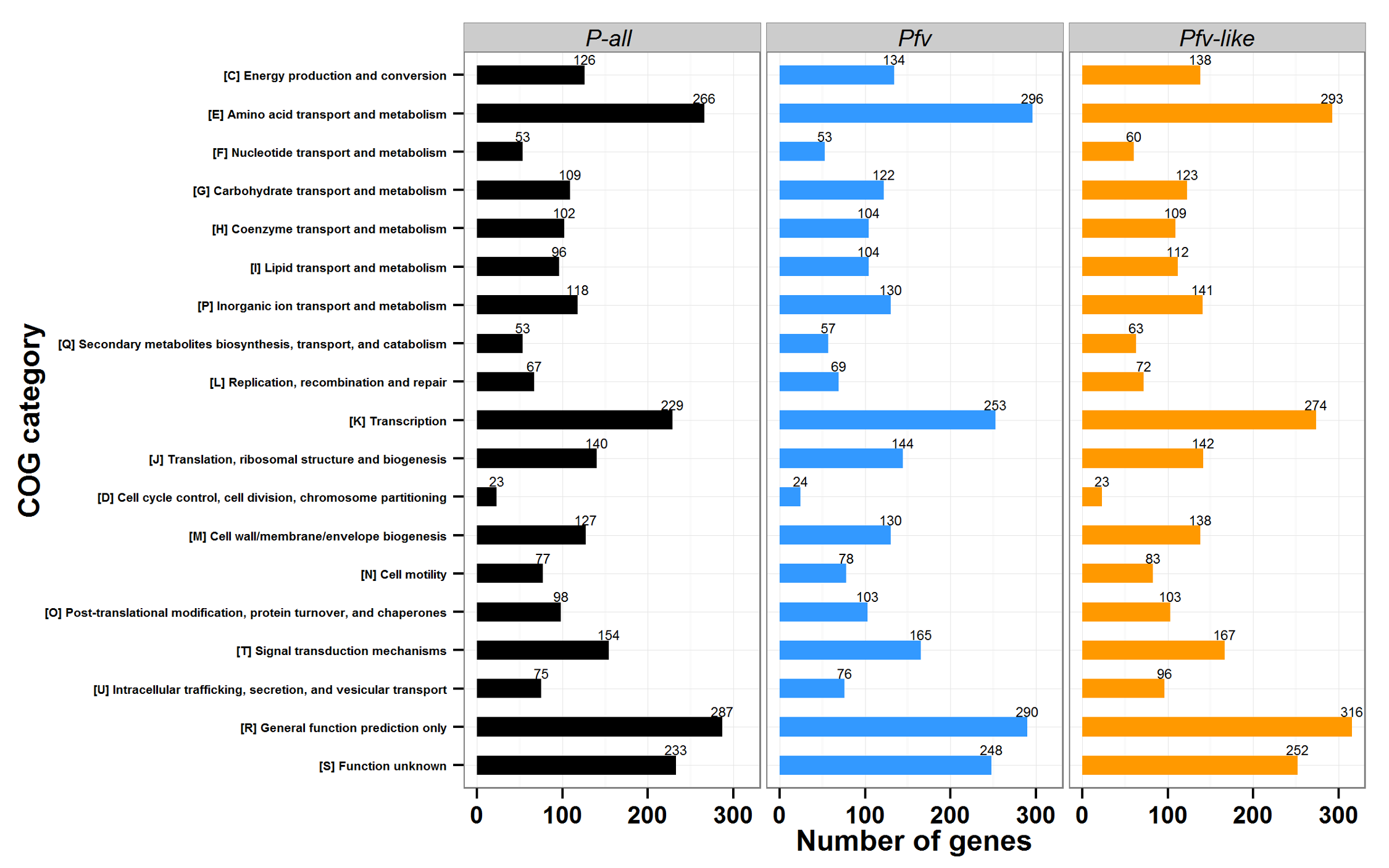

Supplement: S5 Fig — Bar plots representing number of genes in each COG category per rice-infecting Pseudomonas-all (black, n = 8), P. fuscovaginae (blue, n = 5) and P. fuscovaginae-like (orange, n = 3). (TIFF) [file pone.0139256.s005.tiff]
