# Supplementary material for: Rice-Infecting Pseudomonas Genomes Are Highly Accessorized and Harbor Multiple Putative Virulence Mechanisms to Cause Sheath Brown Rot
Source: PLoS One. 2015 Sep 30;10(9):e0139256. doi: 10.1371/journal.pone.0139256 (PMC4589537; doi:10.1371/journal.pone.0139256)
Supplement: S1 Table — The 79 Pseudomonas genomes are listed according to species, strains and their corresponding GenBank ID. (DOCX) [file pone.0139256.s008.docx]

**S1 Table. List of *Pseudomonas* genomes used for comparative analysis in this study.** The 79 *Pseudomonas* genomes are listed according to species, strain name, and corresponding Genbank number.

| **Species** | **Strain** | **Accession Number** |
| --- | --- | --- |
| *Pseudomonas fuscovaginae*-like* | IRRI 6609 | JSYZ00000000 |
| *Pseudomonas fuscovaginae*-like* | IRRI 7007 | JTBY00000000 |
| *Pseudomonas fuscovaginae*-like* | S-E1 | AQOH0100000 |
| *Pseudomonas fuscovaginae** | CB98818 | ALAQ0100000 |
| *Pseudomonas fuscovaginae** | UPB7036 | AIEU00000000 |
| *Pseudomonas fuscovaginae** | DAR 77795 | BATD01000000 |
| *Pseudomonas fuscovaginae** | DAR 77800 | BATE01000000 |
| *Pseudomonas fuscovaginae** | ICMP 5940 | BATG01000000 |
| *Pseudomonas syringae* | BRIP34876 | AMXK01000000 |
| *Pseudomonas syringae* | BRIP39032 | AMZX01000000 |
| *Pseudomonas syringae* | CC94 | AVEA00000000 |
| *Pseudomonas syringae* | CC457 | AVEB00000000 |
| *Pseudomonas syringae* | CC1416 | AVEP00000000 |
| *Pseudomonas syringae* | CC1417 | AVEO02000000 |
| *Pseudomonas syringae* | CC1559 | AVEG00000000 |
| *Pseudomonas syringae* | CC1583 | AVEF00000000 |
| *Pseudomonas syringae* | CC1630 | AVED00000000 |
| *Pseudomonas syringae* | Cit7 | AEAJ00000000 |
| *Pseudomonas syringae* | Lz4W | AOGS00000000 |
| *Pseudomonas syringae* | UB303 | AVDZ00000000 |
| *Pseudomonas syringae* | USA011 | AVDX00000000 |
| *Pseudomonas syringae pv. avellanae* | ISPVe013 | AKCJ01000000 |
| *Pseudomonas syringae pv. avellanae* | ISPVe037 | AKCK01000000 |
| *Pseudomonas syringae pv. glycinea* | B076 | AEGG01000000 |
| *Pseudomonas syringae pv. japonica* | M301072PT | AEAH00000000 |
| *Pseudomonas syringae pv. lachrymans* | M302278PT | AEAM00000000 |
| *Pseudomonas syringae pv. maculicola* | E4326 | AEAK01000000 |
| *Pseudomonas syringae pv. oryzae* | 1-6 | ABZR00000000 |
| *Pseudomonas syringae pv. phaseolicola** | 1448A | AAEZ01000000 |
| *Pseudomonas syringae pv. pisi* | 1704B | AEAI01000000 |
| *Pseudomonas syringae pv. syringae* | B64 | ANZF00000000 |
| *Pseudomonas syringae pv. syringae** | B728a | AABP02000000 |
| *Pseudomonas syringae pv. syringae* | SM | APWT00000000 |
| *Pseudomonas syringae pv. tabaci* | ATCC 11528 | AEAP01000000 |
| *Pseudomonas syringae pv tomato** | DC3000 | AE016853 |
| *Pseudomonas fluorescens** | A506 | CP003041 |
| *Pseudomonas fluorescens** | F113 | NC_016830 |
| *Pseudomonas fluorescens** | Pf0 | AAAT03000000 |
| *Pseudomonas fluorescens** | SBW25 | NC_012660 |
| *Pseudomonas protegens** | Pf-5 | CP000076 |
| *Pseudomonas protegens** | CHA0 | CP003190 |
| *Pseudomonas putida** | BIRD-1 | NC_017530 |
| *Pseudomonas putida** | DOT-T1E | CP003734 |
| *Pseudomonas putida** | F1 | AALM01000000 |
| *Pseudomonas putida** | GB-1 | AAXR01000000 |
| *Pseudomonas putida** | H8234 | NC_021491 |
| *Pseudomonas putida** | HB3267 | CP003738 |
| *Pseudomonas putida** | KT2440 | NC_002947 |
| *Pseudomonas putida** | NBRC 14164 | AP013070 |
| *Pseudomonas putida** | W619 | NC_010501 |
| *Pseudomonas stutzeri** | A1501 | CP000304 |
| *Pseudomonas stutzeri* | ATCC 11728 | CP002881 |
| *Pseudomonas stutzeri* | CCUG 29243 | CP003677 |
| *Pseudomonas stutzeri** | DSM 10701 | CP003725 |
| *Pseudomonas stutzeri* | DSM 4166 | CP002622 |
| *Pseudomonas stutzeri* | RCH2 | CP003071 |
| *Pseudomonas aeruginosa* | B136 33 | CP004061 |
| *Pseudomonas aeruginosa* | DK2 | CP003149 |
| *Pseudomonas aeruginosa* | LES431 | NC_023066 |
| *Pseudomonas aeruginosa* | LESB58 | NC_011770 |
| *Pseudomonas aeruginosa* | M18 | CP002496 |
| *Pseudomonas aeruginosa* | MTB 1 | CP006853 |
| *Pseudomonas aeruginosa* | NCGM2 S1 | NC_017549 |
| *Pseudomonas aeruginosa* | PA1 | CP004054 |
| *Pseudomonas aeruginosa* | PA1R | CP004055 |
| *Pseudomonas aeruginosa** | PA7 | AAQE01000000 |
| *Pseudomonas aeruginosa** | PAO1 | NC_002516 |
| *Pseudomonas aeruginosa* | RP73 | NC_021577 |
| *Pseudomonas aeruginosa* | SCV20265 | CP006931 |
| *Pseudomonas aeruginosa* | UCBPP PA14 | AABQ07000000 |
| *Pseudomonas brassicacearum* | NFM421 | NC_015379 |
| *Pseudomonas denitrificans** | ATCC13867 | CP004143 |
| *Pseudomonas entomophila** | L48 | NC_008027 |
| *Pseudomonas fulva* | 12-X | CP002727 |
| *Pseudomonas mendocina* | NK-01 | CP002620 |
| *Pseudomonas monteilii* | SB3078 | NC_023075 |
| *Pseudomonas monteilii* | SB3101 | NC_023076 |
| *Pseudomonas poea* | RE*1-1-14 | NC_020209 |
| *Pseudomonas resinovorans** | NBRC 106553 | AP013068 |
| ***** Accessions used for phylogenetic tree | | |
