# Supplementary material for: Rice-Infecting Pseudomonas Genomes Are Highly Accessorized and Harbor Multiple Putative Virulence Mechanisms to Cause Sheath Brown Rot
Source: PLoS One. 2015 Sep 30;10(9):e0139256. doi: 10.1371/journal.pone.0139256 (PMC4589537; doi:10.1371/journal.pone.0139256)
Supplement: S4 Table — (DOCX) [file pone.0139256.s011.docx]

**S4 Table.** **Putative pathogenicity related genes found in *P.fuscovaginae*-like genome.**

| **Functional Category** | **Locus ID^a^** | **Number of genes** |
| --- | --- | --- |
| **Type I Secretion System** |  |  |
| *apr* cluster | PF66_01459-01467 | 9 |
| *has* cluster | PF66_03148-03152 | 5 |
| **Type II Secretion System** |  |  |
| *xcp* cluster | PF66_01620-01630 | 11 |
| *hxc* cluster | PF66_04896-04906 | 11 |
| *gsp* cluster | PF66_01113-01123 | 12 |
| **Type III Secretion System** |  |  |
| SPI-1 cluster | PF66_02863-02889 | 26 |
| **Type VI Secretion System** |  |  |
| HSI-1 cluster | PF66_03762-03790 | 29 |
| **Type IV pili** | PF66_01323-01333,03281-03295 | 26 |
| **Flagellum** | PF66_05017-05023,05045-05072 | 34 |
| **Quorum Sensing** |  | 9 |
| PfvI/R | PF66_00570-00572 | 3 |
| PfsI/R | PF66_05829-05831 | 3 |
| Putative quorum sensing | PF66_05967-05970 | 3 |
| **Putative Cell Wall degrading Enzymes** | PF66_02701,04627,04809 | 3 |
| **Secondary metabolite biosynthesis** |  |  |
| auxin | PF66_06080 | 1 |
| achromobactin | PF66_02230-02245 | 16 |
| pyoverdine | PF66_02539-02555 | 17 |
| Putative NRP metabolite | PF66_03119-03143 | 24 |
| Putative NRP metabolite | PF66_03405-03443 | 39 |
| Putative NRP metabolite | PF66_05396-05401 | 6 |

^a^ Locus ID is based on IRRI 6609.
