# Supplementary material for: Rice-Infecting Pseudomonas Genomes Are Highly Accessorized and Harbor Multiple Putative Virulence Mechanisms to Cause Sheath Brown Rot
Source: PLoS One. 2015 Sep 30;10(9):e0139256. doi: 10.1371/journal.pone.0139256 (PMC4589537; doi:10.1371/journal.pone.0139256)
Supplement: S6 Table — (DOCX) [file pone.0139256.s013.docx]

| **Go Identifier** | **Description** | **No. of genes** |
| --- | --- | --- |
| Molecular Function |  |  |
| GO:0005215 | transporter activity | 19 |
| GO:0003824 | catalytic activity | 26 |
| GO:0005488 | binding | 13 |

**S6 Table. Predicted molecular function of the core secreted proteins of rice-infecting *Pseudomonas* using Gene ontology (GO) term.**
